# Molecular imaging of telomerase and the enzyme activity-triggered drug release by using a conformation-switchable nanoprobe in cancerous cells

**DOI:** 10.1038/s41598-018-34670-7

**Published:** 2018-11-05

**Authors:** Hai Shi, Tao Gao, Liu Shi, Tianshu Chen, Yang Xiang, Yuanyang Li, Genxi Li

**Affiliations:** 1 State Key Laboratory of Pharmaceutical Biotechnology and Collaborative Innovation Center of Chemistry for Life Sciences, Department of Biochemistry, Nanjing University, Nanjing, 210093 P. R. China; 2 Center for Molecular Recognition and Biosensing, School of Life Sciences, Shanghai University, Shanghai, 200444 P. R. China; 3 Department of Neurosurgery, Nanjing Integrated Traditional Chinese and Western Medicine Hospital, Affiliated with Nanjing University of Chinese Medicine, Nanjing, 210014 P. R. China

## Abstract

So far, the development of a unique strategy for specific biomolecules activity monitoring and precise drugs release in cancerous cells is still challenging. Here, we designed a conformation-switchable smart nanoprobe to monitor telomerase activity and to enable activity-triggered drug release in cancerous cells. The straightforward nanoprobe contained a gold nanoparticle (AuNP) core and a dense layer of 5-carboxyfluorescein (FAM)-labeled hairpin DNA shell. The 3′ region of hairpin DNA sequence could function as the telomerase primer to be elongated in the presence of telomerase, resulting in the conformational switch of hairpin DNA. As a result, the FAM fluorescence was activated and the anticancer drug doxorubicin (Dox) molecules which intercalated into the stem region of the hairpin DNA sequence were released into cancerous cells simultaneously. The smart method could specifically distinguish cancerous cells from normal cells based on telomerase activity. It also showed a good performance for monitoring telomerase activity in the cytoplasm by molecular imaging and precise release of Dox triggered by telomerase activity in cancerous cells. These advantages may offer a great potential of this method for monitoring telomerase activity in cancer progression and estimating therapeutic effect.

## Introduction

In dividing cancerous cells, telomerase maintains genome integrity by adding repetitive DNA sequences (TTAGGG) to the chromosome ends. About 90% of tumor entities show telomerase activity in dividing cancerous cells^1–3^, in contrast, telomerase activity is rarely detected in normal cells^4–6^. Thus telomerase activity can be considered as an important indicator of cancer progression^7,8^. And this distinct difference has been a focus for cancer mechanism study and therapeutic strategy development^9–11^. Thus, the development of a methodology for monitoring cell-to-cell variation in telomerase activity from different samples is urgently needed in biomedical research and clinical practice.

Hitherto, a variety of methods have been developed to investigate the telomerase activity by using various techniques, such as fluorescence^12–15^, electrochemistry^16–19^, surface enhanced Raman scattering^20^, surface plasma resonance^21,22^, colorimetry^23–25^, chemiluminescence^26^, and electrochemiluminescence^27,28^. However, these technologies can only analyze the bulk samples *in vitro*, such as cell extraction and urine^12,14^. They cannot enable us to give insight into telomerase level in complex biological environments, particularly in living cells. Observation of biomolecules in living cells is fundamental to a quantitative understanding of how biological systems function^29^. So, developing an approach for quantitative and precise detection of telomerase activity in living cells will be of help to understand its function within cancerous cells and its application in the diagnosis of disease.

It has come to light that molecular fluorescence imaging can reveal the activity of biomolecules of interest in living cells^30–32^, which can be an efficient way to reflect the intracellular activity of telomerase, and also indicate activity change caused by cellular activity^33^. More recently, several studies are focused on the fluorescence imaging of telomerase based on DNA-functionalized nanoprobes^34–38^. In these studies, flexible DNA nanostructures are engaged in the detection of telomerase activity. However, some nonnegligible disadvantages remained. The synthesis of these probes needs one or multiple custom-built DNAs to fabricate highly structured nanoprobes. Such structures may not be suitable for practical application, because they may result in unnecessary dissociation of DNA complexes formed by two or more different single-strand DNAs during cellular uptake. Besides, the nanoprobes assembled by heterogeneous DNA hybridization will be challenged by DNA polymerase, causing strand displacement during DNA amplification. These limitations may lead to false positive in complex environment of a cell. To mitigate these limitations, here we simplified the design and construction of the nanoprobes by introducing a rational designed hairpin DNA. The functionalized nanoprobe can be responsive to telomerase activity-triggered DNA conformation switch, while avoiding the interference of polymerase in cells and lowering false positive in complex environment of a cell.

In our design, the nanoprobe is composed of a gold nanoparticle (AuNP) core and a shell with a dense layer of hairpin DNAs. These hairpin DNAs are covalently linked to gold nanoparticles (AuNPs) through Au-S bonds. The 3′ region of hairpin DNA sequence is designed as a primer that could be elongated by telomerase, and the detailed sequences are summarized in Table S1. The fluorophore FAM modified in the stem part of hairpin DNA is quenched as a result of proximity to AuNP surface. In the presence of telomerase, the primer is elongated to produce telomeric repeats (TTAGGG), which trigger the competitive hybridization with the stem part of hairpin DNA. As a result, the 5′ region of hairpin DNA sequence is substituted by the extension chain, giving rise to subsequent DNA conformational switch. The DNA conformational switch produces new hairpin DNA to make the fluorescence of FAM recovered. To improve the performance of the nanoprobe, a four-base pairs of DNA is designed into the 3′ terminal region of hairpin DNA sequence, which could facilitate the DNA conformational change in the presence of telomerase. Therefore, our proposed method can reveal the telomerase activity in the cytoplasm through the conformational switch of hairpin DNA in cancerous cells. On the basis of the obvious difference in telomerase activity between cancerous cells and normal cells, telomerase activity-responsive drug carriers will facilitate more precise and easily controlled drug release than the conventional stimuli-driven drug release system^34,36,39^. Thus, we have further used the nanoprobe to load Dox molecules via GC base pairs-Dox interaction^40^ and the telomerase activity-triggered conformation switch has been observed to be able to induce specific release of trapped Dox molecules into cancerous cells.

## Results and Discussion

### Design principle of telomerase activity-responsive AuNP-based molecular probes (AuNP-MPs) and drug release

**Figure 1:**
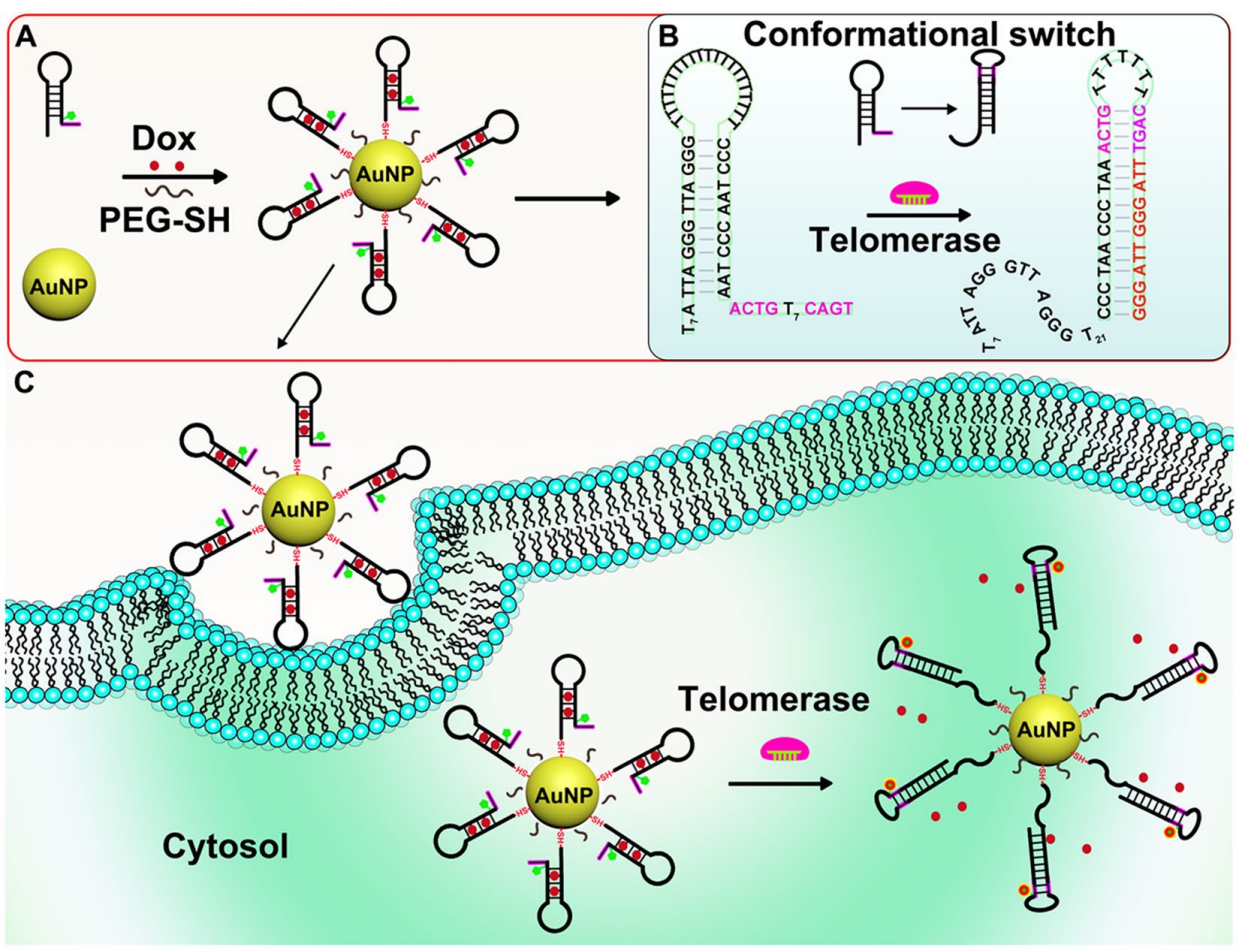
Schematic illustration of telomerase activity monitoring and drug release based on DNA conformational switch triggered by telomerase activity in living cells. (**A**) The synthesis procedure of AuNP-MPs. (**B**) The detailed conformational switch process. (**C**) The intracellular imaging and drug release based on DNA conformational switch initiated by telomerase activity.

In this work, we proposed a straightforward method by using a conformation-switchable smart nanoprobe for telomerase imaging in cancerous cells and telomerase activity-responsive Dox release. Its architecture and design mechanism were illustrated in Fig. 1. In detail, the nanoprobe was composed of an AuNP core and a dense layer of hairpin DNAs (HP-1) (Fig. 1A). The HP-1 was conjugated on AuNPs via Au-S bonds at 5′ end of the sequence. Due to the stem-loop structure of HP-1, FAM modified in the stem of HP-1 could be close to the surface of AuNPs, resulting in the fluorescence quenching. Dox molecules were inserted into the GC base pairs of the stem part, their fluorescence were quenched because of GC base pairs-Dox interaction. As illustrated in Fig. 1B,C, in the presence of telomerase, the free 3′ region of HP-1, which was not hybridized with 5′ region of HP-1, would function as the primer of telomerase to be elongated to generate telomeric repeats. The elongated telomeric repeats could competitively hybridize with the stem part of HP-1. As a result, the 5′ region of HP-1 was substituted by telomeric repeats and the loop was opened subsequently. This substitution process triggered the conformational switch of HP-1 to form a new hairpin DNA. As the most direct effect, the quenched FAM was lighted up and the trapped Dox molecules were released into cancerous cells for specific telomerase activity monitoring and precise Dox release.

To enhance the stability and reduce the immunogenicity of Dox-loaded AuNP-MPs (Dox-AuNP-MPs), thiolated polyethylene glycol (PEG-SH) was modified on AuNPs. The performance of the AuNP-MPs was improved by linking a four-base pairs of DNA with the 3′ terminal region of HP-1, as shown in Fig. 1B. A four-base pairs of DNA was expected to form an intramolecular toehold for facilitating the DNA conformational change after the primer elongation. Specifically, the intramolecular toehold was a short DNA sequence (ACTG), which could form an unstable small hairpin DNA in the absence of telomerase. Upon addition of telomerase, the toehold sequence could first act as the primer to be elongated by telomerase, and then the formation of small hairpin DNA produced an intramolecular toehold to further enhance the competitive hybridization of the elongated telomeric repeats with the stem part of the HP-1.

### Characterization of AuNP-MPs

The AuNP-MPs for telomerase activity monitoring were successfully synthesized and characterized (Fig. S1). After AuNPs were functionalized with HP-1, the typical absorption peak of AuNPs in the UV-vis absorption spectra was red-shifted from 520 nm to 523 nm (curve a, b, Fig. S1), and the characteristic peak of DNA at 260 nm appeared compared to unmodified AuNPs. Furthermore, the increased absorption value at 260 nm was observed after telomerase elongation compared to that of AuNP-MPs in the absence of telomerase (curve c, Fig. S1). The average diameter of AuNP-MPs characterized via TEM was approximately 13 nm, which was similar to that of bare AuNPs (Fig. S2A,B). But the modification of PEG made the AuNP-MPs with higher dispersibility than bare AuNPs, because PEG as an electrically neutral ligand effectively restrained the aggregation of AuNP^41–43^. As illustrated in Fig. S1C,D, dynamic light scattering data showed that the hydrodynamic diameter of AuNP-MPs was 38.3 nm, which of AuNPs was 21.9 nm, indicating that the surface load increased the diameter of nanoprobe. These results suggested that the hairpin DNA were successfully linked on the surface of AuNPs. In addition, the amount of hairpin DNA loaded on each AuNP was estimated to be about 27 (Fig. S3).

### The feasibility of the proposed strategy and its condition optimization

**Figure 2:**
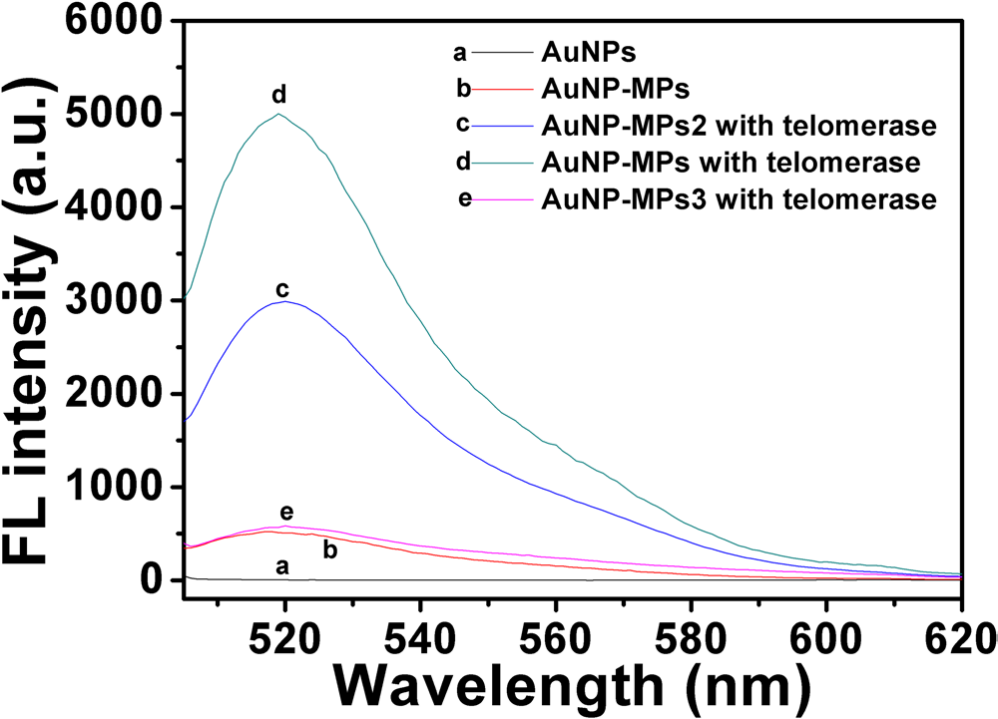
Fluorescence response of bare AuNPs (**a**), HP-1 (with toehold)-modified AuNPs (AuNP-MPs) in the absence of telomerase (**b**), HP-2 (without toehold)-modified AuNPs (AuNP-MPs2) after incubation with telomerase for 2 h (**c**), HP-1 (with toehold)-modified AuNPs (AuNP-MPs) after incubation with telomerase for 2 h (**d**) and HP-3-modified AuNPs (AuNP-MPs3) after incubation with telomerase for 2 h (**e**).

Once we validated that the hairpin DNA was successfully conjugated on AuNPs, we sought to test whether the prepared AuNP-MPs could make expectative response to telomerase activity. But before that, telomeric repeat amplification protocol (TRAP) assay was performed to validate the feasibility of HP-1 as primer of telomerase, as a positive control, TS primer was also used for telomerase substrate. As a result, the 6-bp incremental ladder produced by various concentrations of telomerase could be visualized in gel (Fig. S4A). In order to further verify that these incremental ladders were indeed associated with telomerase activity, TRAP assay was performed by incubating with the epigallocatechin gallate (EGCG)-treated cell extracts, since EGCG is a well-characterized telomerase inhibitor. The results showed that the incremental ladder and the brightness of bands decreased with the increasing concentration of EGCG (Fig. S4B). So, the results showed that HP-1 could be indeed used as telomerase substrate, and the telomerase activity could be inhibited by EGCG. Next, the response of AuNP-MPs to telomerase activity was tested, as shown in Fig. 2. Bare AuNPs had no observable fluorescence signal in 1× TRAP reaction buffer (curve a, Fig. 2). The AuNP-MPs were incubated with dNTPs in 1× TRAP reaction buffer at 37 °C in the absence of telomerase, which exhibited no obvious fluorescent signal, suggesting that the FAM fluorescence was effectively quenched by the AuNPs (curve b, Fig. 2). Upon the addition of telomerase extraction, the significant enhancement in fluorescence intensity was observed (curve d, Fig. 2). The results confirmed that telomerase activity-induced fluorescence switch based on intramolecular conformational switch of HP-1 was successfully constructed. In our work, we introduced a four-base pairs of toehold to enhance the response of AuNP-MPs to telomerase activity. Next, to verify the actual effect of the design, four bases were deleted to form a shortened hairpin DNA (HP-2) which was lack of toehold. HP-2-modified AuNPs (AuNP-MPs2) were incubated with the telomerase extraction, and then 40% decrease in the fluorescence intensity than that of AuNP-MPs with the intramolecular toehold was observed (curve c, Fig. 2). The result suggested that the introduction of toehold improved the release of FAM from the AuNPs surface, in other words, more hairpin DNA sequences performed the DNA chain exchanges with the help of toehold. On the contrary, as a control, HP-3 was designed to construct AuNP-MPs3, which could not be triggered to induce conformational change due to lack of complementary sequence of telomeric repeats (TTAGGG) and intramolecular toehold (ACTG) (curve e, Fig. 2). In view of above results, the HP-1 containing an intramolecular toehold was used in the subsequent experiments.

To obtain the optimal detection performance, the incubation time was investigated. As illustrated in Fig. S5, a gradual increase of the fluorescence intensity of AuNP-MPs with an increasing incubation time was observed, and the fluorescence intensity plateaued after 2 h. Thus, the incubation time for telomerase elongation reaction was chosen to be 2 h.

### The stability of AuNP-MPs

The next experiments were performed to test the stability of the prepared AuNP-MPs against damage by intracellular enzymes and reductants. DNase I, T4 DNA ligase, GSH, DNA polymerase I and DTT were individually incubated with AuNP-MPs. Both GSH and T4 DNA ligase had almost no effect on the fluorescent signal (Fig. S6A). More remarkable, the results also manifested that the HP-1 conjugated on the surface of AuNPs were not degraded by DNase I. But, by contrast, on addition of the telomerase extraction or the strong reductant DTT, obvious fluorescence recovery was detected. Unlike DNA conformational change caused by telomerase elongation, the fluorescence recovery resulted from DTT treatment was attributed to the break of the Au-S bond and detachment of DNA from AuNPs surface^44^. To test the performance of the synthesized nanoprobe against DNA polymerase, DNA polymerase I (Klenow fragment) was chosen as a model. After incubation with DNA polymerase I for 2 h in the presence of dNTPs, the fluorescence intensity of AuNP-MPs had little increase than that of AuNP-MPs without dNTPs, which may be caused by DNA polymerase I elongation (Fig. S6B). After 12 h, the fluorescence intensity of AuNP-MPs increased further regardless of the dNTPs change. The results suggested that the fluorescence increase after 12 h were not due to the strand displacement caused by DNA polymerase elongation, it was speculated to be caused mainly by the DTT in the DNA polymerase. The DTT was added to protect DNA polymerase by the manufacturer. Therefore, according to the above results, the fluorescence intensity increase caused by DNA polymerase I could be ignored during telomerase activity monitoring. The stability of AuNP-MPs was also confirmed in different media, such as 10% FBS, DMEM with 10% FBS, PBS and whole blood (Fig. S7). In summary, these results suggested that the telomerase-specificity and structural stability of AuNP-MPs, showed the favorable for telomerase detection and cellular applications.

### Capability investigating of AuNP-MPs for telomerase activity detection

**Figure 3:**
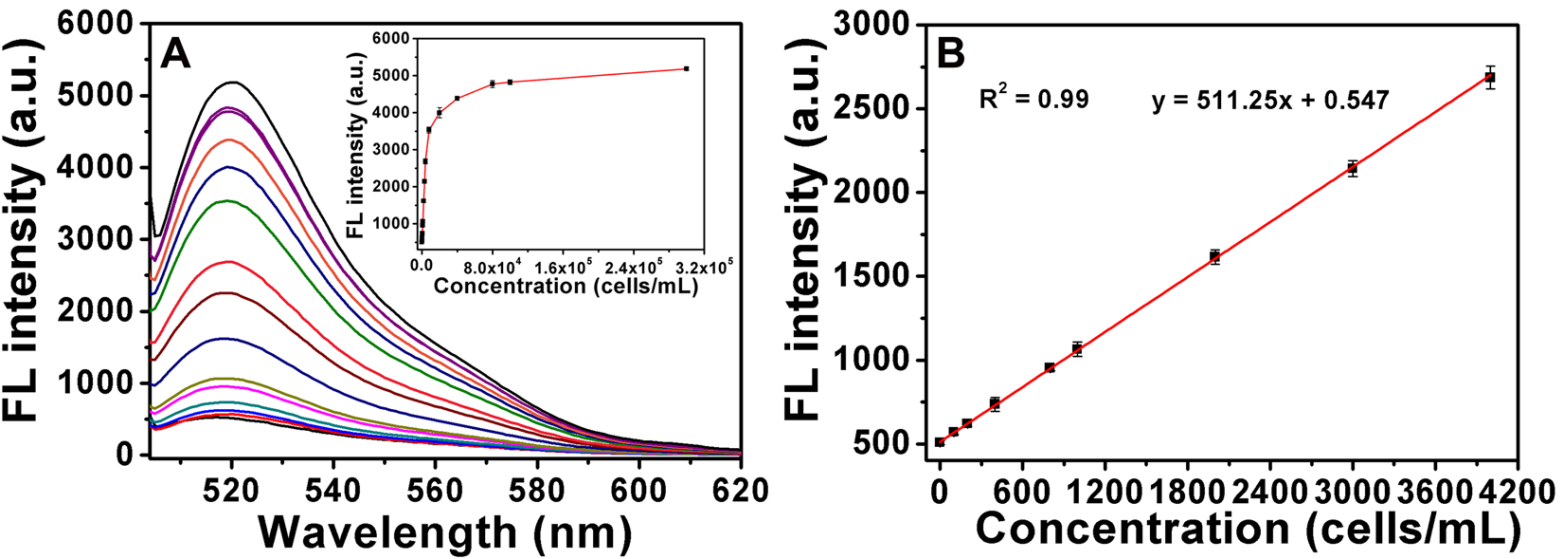
(**A**) Fluorescence emission spectra of 4 nM AuNP-MPs after incubation with various concentrations of HeLa cell extracts for 2 h. The telomerase extracts come from different concentrations of HeLa cells, which are from 0 to 3 × 10^5^ cells/mL. Inset in A: the fluorescence intensity of AuNP-MPs at emission wavelength of 520 nm in response to different concentrations of extracts. (**B**) The relationship between the fluorescence intensity and HeLa cells with ranging from 0 to 4000 cells/mL. Error bars show the standard deviations of three experiments.

The validity of the designed AuNP-MPs for telomerase activity analysis was evaluated in following work. In optimized condition, we measured the fluorescence intensity changes in response to the telomerase extracts from various concentrations of HeLa cells. As expected, higher concentration of the telomerase extract led to larger fluorescence increase, which wonderfully verified our design. A linear relationship was observed between the fluorescence intensity and telomerase extracts from 0 to 4000 cells/mL, as demonstrated in Fig. 3. The regression equation was calculated to be y = 511.25 × +0.547, the correlation coefficient (R^2^) of the calibration curve was 0.99. According to 3σ/k (σ, the standard deviation of the blank sample, k, the slope of standard curve), the detection limit was calculated to be 59 cells/mL. According to the reported telomerase activity in one HeLa cell, 3.1 × 10^−11^ IU^39^, the detection limit of our method was evaluated to be 1.83 × 10^−9^ IU/mL.

### *In situ* imaging of intracellular telomerase activity

**Figure 4:**
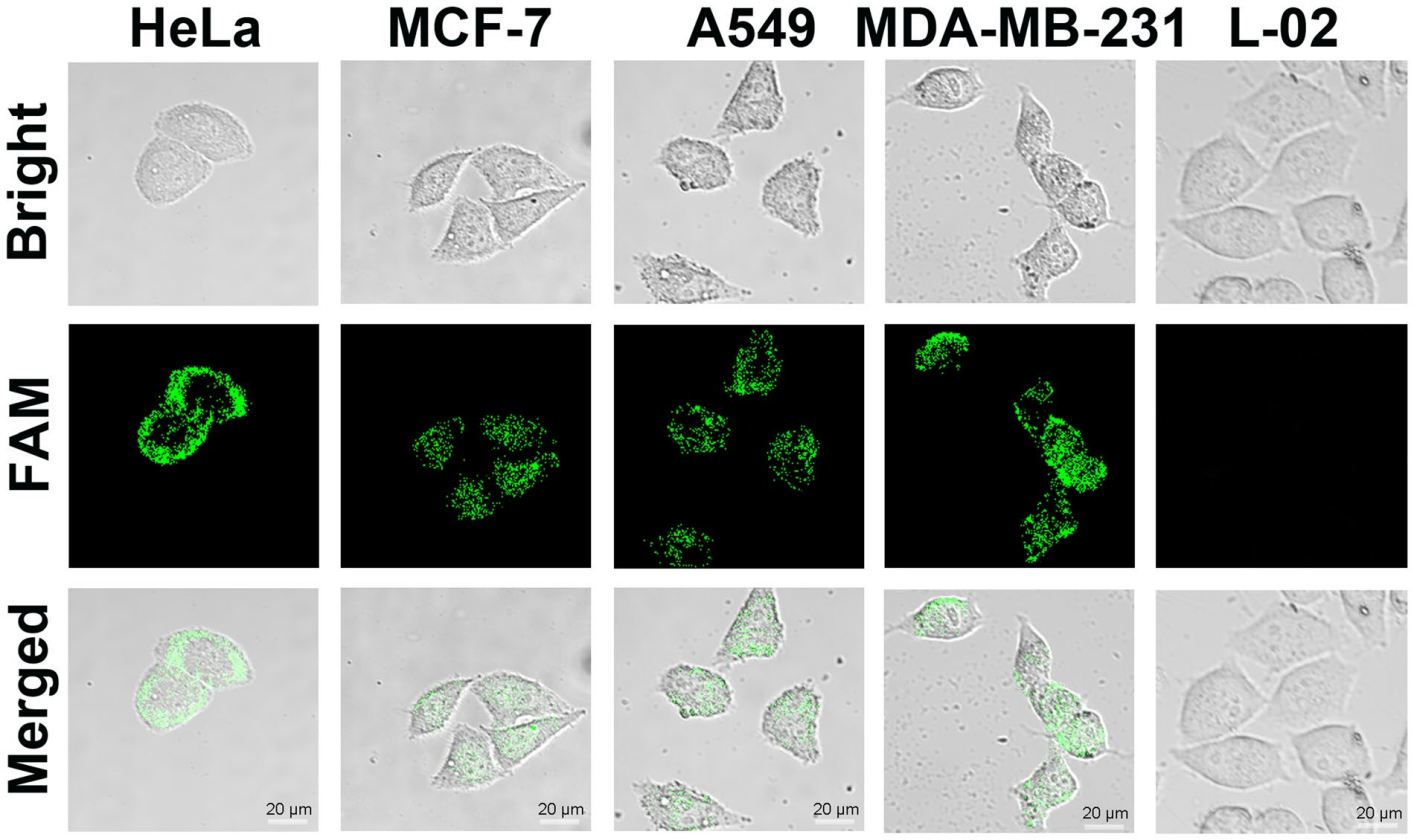
Confocal images for evaluation of DNA conformational switch-based AuNP-MPs for telomerase activity monitoring in individual cells: HeLa, MCF-7, A549, MDA-MB-231, and L-02 cells.

It is of great importance for cancer early diagnosis and therapy through evaluating the telomerase activity in living cells. Therefore, based on the results above, we further utilized this AuNP-MPs to monitor the telomerase activity in several different cancer cells, including HeLa (human cervical cancer cell line), MCF-7 (human breast cancer cell line), A549 (human lung adeno carcinoma epithelial cell line), MDA-MB-231 (human breast cancer cell line), and L-02 (normal human hepatocyte cell line). First of all, to obtain the optimal imaging results, the AuNP-MPs were incubated with HeLa cells for different time periods. The confocal images revealed that the fluorescence intensity inside the cells increased with the prolonged incubation time (Fig. S8). After incubating with HeLa cells for 2 h, the fluorescent signal reached the maximum. For this reason, 2 h of incubation time was further used for cellular imaging in the subsequent experiments. Importantly, these images revealed that the prepared AuNP-MPs could be efficiently internalized and sensitively responded by HeLa cells. The verified results are the prerequisite for telomerase detection and drug delivery in cancerous cells. Subsequently, the confocal images of the AuNP-MPs internalized into MCF-7, A549, MDA-MB-231, and L-02 cells were acquired via the confocal microscopy. As indicated in Fig. 4, all cancer cell lines demonstrated distinct telomerase activity, but no telomerase activity was found in the L-02 cells. The distinguishing fluorescence intensity between cancerous and noncancerous cells signified that the AuNP-MPs were successfully lighted up because of the DNA conformational switch induced by telomerase activity in cancerous cells. Due to the nuclear membrane barrier function, our designed nanoprobes can hardly be internalized into nucleus without a nuclear localization signal, especially, in a short time. So the green fluorescence was observed predominantly and even exclusively in the cytoplasm of HeLa cells (Fig. 4). The flow cytometric analysis was performed to detect the fluorescence signal of the AuNP-MPs in different cells, which was similar to the confocal images (Fig. S9A). To exclude the possibility that differences in fluorescence signal was caused by AuNP-MPs uptake, the inductively coupled plasma optical emission spectroscopy (ICP-OES) was used to quantify the relative amount of AuNP-MPs in each cell, as shown in Fig. S10A. As a result, HeLa, MCF-7 and L02 cells showed similar uptake of AuNP-MPs after incubation for 2 h. So, the differences in fluorescence intensity between cancer cells and L-02 cells were associated with telomerase activity rather than the total amount of AuNP-MPs inside the cells. Therefore, the AuNP-MPs exhibit a promising potential for monitoring telomerase activity in living cells and distinguishing cancerous cells from normal cells.

**Figure 5:**
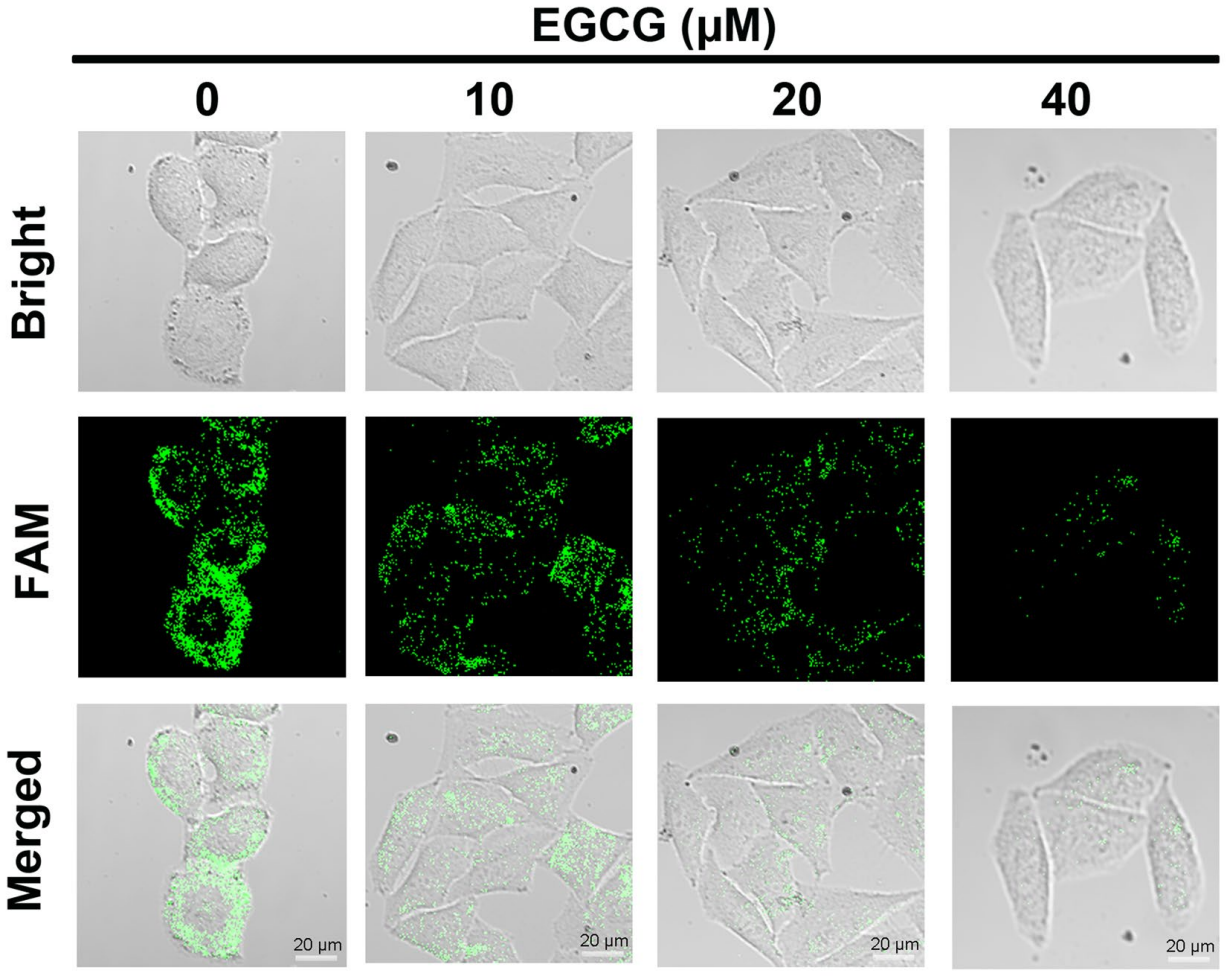
Confocal images of HeLa cells after incubation with different concentrations of EGCG (0 μM, 10 μM, 20 μM, or 40 μM) for 24 h, and then incubated with 100 μL 1.2 nM AuNP-MPs for 2 h.

To confirm that the fluorescence recovery in cells was indeed due to telomerase activity, we incubated HeLa cells with various levels of EGCG. The results were demonstrated in Fig. 5, which showed the fluorescence intensity gradually reduced with increasing concentrations of EGCG. The conclusion was also proved via TRAP assay (Fig. S4B) and flow cytometry (Fig. S8B). These results confirmed that the AuNP-MPs were specific to telomerase activity, which could be used to precisely reflect telomerase activity in cancerous cells.

### *In situ* imaging of intracellular telomerase activity-triggered Dox release

**Figure 6:**
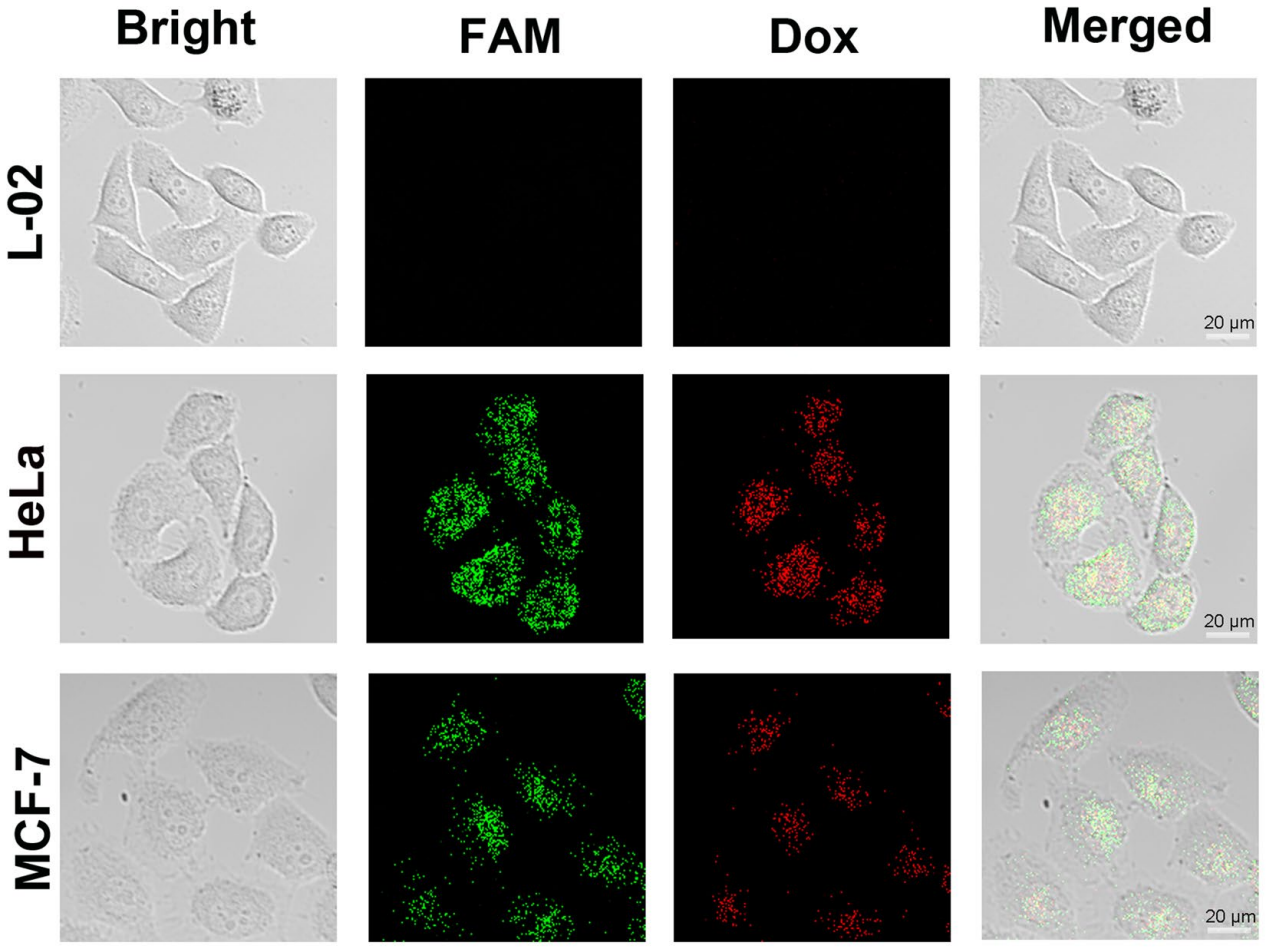
The evaluation of DNA conformational switch-based Dox-AuNP-MPs for telomerase activity monitoring and Dox delivery in individual cells: HeLa, MCF-7 and L-02 cells.

Considering the efficient cellular uptake of AuNP-MPs, we further constructed a telomerase activity-initiated drug release nanocarrier by intercalating Dox molecules into the GC base pairs of the stem of HP-1. The fluorescence of Dox could be quenched after inserting into HP-1. Upon hairpin structure was unfolded because of DNA conformational switch caused by telomerase elongation, the Dox molecules could be released into cancerous cells. It was calculated that approximately 154 Dox molecules could be loaded on one AuNP-MP (Fig. S11). The design was verified through *in vitro* experiments (Fig. S12), the fluorescence intensity of the released Dox from the hairpin DNA was enhanced with the increasing reaction time of telomerase elongation. The recovered fluorescence of Dox was consistent with that of FAM. Furthermore, as shown in Fig. S13, higher concentration of the telomerase extract led to higher Dox fluorescence increase, indicating more Dox release from Dox-AuNP-MPs, and reached equilibrium at 1 × 10^5^ cells/mL. On the contrary, little fluorescence increase could be observed in Dox-AuNP-MPs3 with increasing concentration of telomerase extract. These results confirmed the Dox could be indeed released from the Dox-AuNP-MPs at the moment of DNA conformation switch. Exactly, in the cellular experiments, we observed that the Dox molecules were released into the cancer cells, as shown in Fig. 6. Furthermore, the fluorescence intensity of released Dox was gradually increased with the increase of incubation time (Fig. S14), which was consistent with the FAM signal. ICP-OES analysis has showed the relative amount of Au was almost the same in different cells, which validated the difference in fluorescence intensity of Dox was indeed caused by the difference of telomerase activity (Fig. S10B). In addition, the extent of Dox release was dependent on telomerase activity, which was confirmed by the incubation of Dox-AuNP-MPs and EGCG-treated HeLa cells. Actually, the fluorescence intensity of FAM and Dox gradually reduced with the increasing concentrations of EGCG (Fig. S15). These results indicated that the synthesized Dox-AuNP-MPs were also utilized to the controlled release of Dox triggered by telomerase other than monitoring telomerase activity in living cells.

### The tumor inhibition efficacy of the Dox-AuNP-MPs

The next topic of interest was the tumor inhibition efficacy of the Dox-AuNP-MPs. To examine the tumor inhibition efficacy, MTT assay was conducted on HeLa and L-02 cells (Fig. S16). No obvious decrease of cell viability was found in L-02 cells in AuNPs, AuNP-MPs, or Dox-AuNP-MPs-treated groups, compared to the free Dox group. By contrast, in HeLa cells, Dox or Dox-AuNP-MPs treatment resulted in a significant reduction in cell survival, which was due to telomerase-specific Dox release into HeLa cells from Dox-AuNP-MPs. From another aspect, the above results also suggested that the cytotoxicity of AuNPs and AuNP-MPs on cells were very little. Therefore, the cell MTT assay demonstrated the specific and precise cancerous cell inhibition of Dox-AuNP-MPs.

## Conclusions

In this study, we have successfully synthesized a smart nanoprobe, which could be responsive to telomerase activity-triggered intramolecular DNA conformation switch specifically. The nanoprobe could be simply designed and constructed. The nanoprobe could also be used for the detection of telomerase activity of HeLa cell extracts *in vitro*, the LOD of which was comparable to the previous reports (Table S2). By taking advantages of the significant merits of the AuNP-MPs, we have successfully used the AuNP-MPs to monitor the telomerase activity *in vivo* with cancerous cells-specific targeting. This smart nanoprobe is functionalized with the intramolecular DNA conformation switch structure, which could be avoid effectively the interference of polymerase in cells, lower false positive in complex environment, and showed a good stability in different medium. As a drug nanocarrier, it efficiently releases drug to cancerous cells that is caused by telomerase activity. Given these advantages, the straightforward strategy based on AuNP-MPs shows a promising potential for aiding in specific cancerous cells recognition and reducing the undesired death of healthy cells that is commonly confronted in conventional chemotherapy.

## Experimental Section

### Materials and reagents

Chloroauric acid tetrahydrate (HAuCl_4_·4H_2_O, 99.99% purity) was purchased from Alfa Aesar Co., Inc. (MA, USA). Sodium citrate dihydrate (Na_3_C_6_H_5_O_7_·2H_2_O, 99% purity), sodium chloride (NaCl), glycerol and potassium chloride (KCl) were obtained from Nanjing Chemical Reagent Co., LTD. (Nanjing, China). Trismetyl aminomethane (Tris), magnesium chloride (MgCl_2_), Tween-20, tris(2-carboxyethyl)phosphine (TCEP), ethylene glycol tetraacetic acid (EGTA), 3-[(3-cholamidopropyl)dimethylammonio]-1-propanesulfonic acid (CHAPS), phenylmethylsulfonyl fluoride (PMSF), dithiothreitol (DTT), and epigallocatechin gallate (EGCG) were purchased from Sigma-Aldrich Inc. (St. Louis, USA). T4 DNA ligase, DNA polymerase I (Klenow fragment) and deoxynucleotide (dNTP) solution mix were obtained from New England BioLabs (Ipswich, USA). PEG1000-SH was purchased from Shanghai Ponsure Biotechnology Co., Ltd. (Shanghai, China). MTT cell proliferation and cytotoxicity assay kit, DNase I and doxorubicin hydrochloride (Dox) were purchased from Sangon Biotech (Shanghai) Co., Ltd. (Shanghai, China). All other chemicals used in this work were of analytical grade without further purification. The ultrapure water with a resistivity of 18.2 MΩ·cm was produced by using a Milli-Q apparatus. (Millipore Co., USA). All oligonucleotides used in this work were synthesized and HPLC purified by Sangon Biological Engineering Technology & Co. Ltd (Shanghai, China).

### Synthesis of gold nanoparticle-based molecular probes (AuNP-MPs)

AuNPs with an average diameter of 13 nm were prepared by citrate reduction method^45,46^. Briefly, 4 mL sodium citrate solution (19.4 mM) was added rapidly into a boiling 40 mL HAuCl_4_ solution (0.5 mM) with vigorous stirring. The reaction maintained 30 min until the color of the solution gradually turned to deep red. The prepared AuNPs solution was naturally cooled to room temperature and the final product was stored at 4 °C. The hairpin DNA was heated at 90 °C for 5 min, and slowly cooled down to room temperature for more than 1 h to ensure complete formation of hairpin structure before use. Next, 100 µL 10 µM hairpin DNA was treated with 1 mM TCEP to activate the thiol-modified DNA. After 1 h, the prepared DNA solution was mixed with 6 nM AuNPs solution with the concentration ratio of 100: 1 and incubated overnight for more than 16 h at room temperature. The following day, PEG-SH (120 µM) was added into the mixture and incubated for 2 h. 2 M NaCl was added to reach a final salt concentration of 0.2 M with a stepwise process. After 24 h, to remove the excess hairpin DNA, the solution was centrifuged at 12000 rpm for 30 min and then washed twice with Tris-HCl (10 mM Tris, 10 mM NaCl, pH 7.5). Finally, the synthesized AuNP-MPs were re-dispersed in Tris-HCl and stored at 4 °C for use.

### Synthesis of Dox-loaded AuNP-MPs (Dox-AuNP-MPs)

To prepare Dox-AuNP-MPs, 20 µM Dox was incubated with the AuNP-MPs in Tris-HCl and shaken overnight. The mixture was centrifuged at 14000 rpm for 10 min and washed twice with Tris-HCl, then re-dispersed in Tris-HCl for the next experiments.

### Characterization of the synthesized nanoprobes

The morphologies of the synthesized AuNPs and AuNP-MPs were acquired with an H-7650 transmission electron microscopic (Hitachi, Japan). The particle sizes of AuNPs and AuNP-MPs were characterized by a Zetaplus/90plus dynamic light scattering instrument (Brookhaven Instrument Co., USA). The ultraviolet-visible (UV-vis) spectra were recorded using a Shimadzu UV-1800 spectrometer (Shimadzu Inc., Kyoto, Japan).

### Quantitation of hairpin DNA number on one AuNP

The AuNP-MPs were prepared according to the above synthesis procedures. Then 200 μL AuNP-MPs (2 nM) were treated with 20 mM DTT and incubated together for 12 h under shaking at room temperature. The mixture was centrifuged at 14000 rpm for 10 min, and the supernatant was collected for fluorescence measurement with a Hitachi F-7000 fluorescence spectrophotometer at the emission wavelength of 520 nm. To quantitate the number of hairpin DNA on one AuNP, the fluorescence of a dilution series of hairpin DNA with known concentrations was detected to generate standard curve.

### Quantitation of Dox molecules loaded on the AuNP-MPs

20 µM Dox was incubated with prepared AuNP-MPs and shaken overnight. The mixture was separated to obtain the supernatant, subsequently its fluorescence intensity was measured with a fluorescence spectrometer. Under the same condition, a standard linear calibration curve of Dox was generated. Finally, the molar concentration of Dox could be converted by the fluorescence intensity.

### Cell culture and telomerase extraction

HeLa, MCF-7, MDA-MB-231, and A549 cells were cultured in Dulbecco’s modified Eagle’s medium (DMEM, Gibco) containing 10% fetal bovine serum (FBS, Gibco), penicillin (100 µg/mL), and streptomycin (100 µg/mL). L-02 cells were cultivated in Roswell Park Memorial Institute 1640 medium (RPMI 1640, Gibco) supplemented with 10% FBS, penicillin (100 µg/mL), and streptomycin (100 µg/mL). All cells were maintained at 37 °C in a humidified atmosphere with 5% CO_2_.

To extract telomerase, cells were collected during the exponential phase of growth. Cells were dispersed in a 1.5-mL EP tube and rinsed twice with ice-cold PBS solution (10 mM Na_2_HPO_4_, 2 mM NaH_2_PO_4_, pH 7.4) spiked with 135 mM NaCl, 4.7 mM KCl. Next, cells at a concentration of 3 × 10^6^ cells/mL were re-suspended in 200 μL of ice-cold CHAPS lysis buffer (10 mM Tris-HCl, pH 7.5, 1 mM MgCl_2_, 1 mM EGTA, 0.5% (W/V) CHAPS, 10% (V/V) glycerol) and incubated on ice for 30 min. Subsequently, the lysate was centrifuged at 12000 rpm at 4 °C for 20 min to collect the supernatant. Finally, the upper lysate solution was carefully transferred to a fresh RNase-free tube and stored at −80 °C before use.

### TRAP assay

0.5 μM HP-1 or TS primer was first extended by incubating with different concentration of telomerase extracts in the presence of 250 μM dNTPs at 37 °C. After 2 h, the extension products were amplified by PCR in the presence of CX primer: 95 °C for 5 min to inactivate telomerase, and 40 cycles at 95 °C for 30 s, 52 °C for 30 s and 72 °C for 30 s. Following PCR, 2 μL GelRed dye was added to each TRAP reaction mixture and run on a 12% nondenaturing acrylamide gel in 1× TBE. To validate EGCG-induced telomerase activity inhibition, HP-1 was incubated with different concentration of EGCG-treated telomerase extract, and then PCR experiment was performed with same conditions.

### Fluorescent detection of telomerase activity in cell extract

Telomerase activity detection of HeLa cell extract was performed by mixing variable amounts of cell extract with 4 nM AuNP-MPs in the presence of 400 μM dNTPs. After incubating at 37 °C for 2 h, the fluorescence intensity of the mixture was detected under excitation at 493 nm.

### Investigation of the stability of AuNP-MPs

20 mM DTT, 0.4 mg/mL DNase I, 4 U/mL T4 DNA ligase, and 15 mM GSH were mixed with AuNP-MPs (4 nM), respectively. After incubation for 12 h in 200 μL 1× TRAP reaction buffer containing 20 mM Tris-HCl (pH 8.3), 1.5 mM MgCl_2_, 63 mM KCl, 0.005% (V/V) Tween-20, and 1 mM EGTA, the fluorescence intensity of these mixtures was collected with a 493 nm excitation wavelength, respectively. Together, for comparison, 20 μL telomerase lysis solution was also incubated with AuNP-MPs (4 nM) at 37 °C for 2 h.

### Investigation of the AuNP-MPs against DNA polymerase

DNA polymerase I (Klenow fragment) was chosen as a model to test the stability of AuNP-MPs against DNA polymerase. 4 U/mL DNA polymerase I was incubated with 4 nM AuNP-MPs at 37 °C in Tirs-HCl (10 mM Tris, 10 mM MgCl_2_, 50 mM NaCl, pH 7.9) in the presence or absence of 400 μM dNTPs. The fluorescence intensity was detected with a 493 nm excitation wavelength.

### Release of Dox from telomerase activity-responsive Dox-AuNP-MPs

Dox-AuNP-MPs (4 nM) were mixed with 400 μM dNTPs in 1× TRAP reaction buffer and incubated at 37 °C for 2 h after addition of different concentration of telomerase extraction to obtain the fluorescence intensity of Dox.

### Intracellular imaging of telomerase activity with AuNP-MPs

HeLa, MCF-7, A549, MDA-MB-231, and L-02 cells (1 mL, 1 × 10^5^ cells/mL) were seeded into a 15 mm confocal dish and incubated for 24 h, respectively. 100 μL 1.2 nM AuNP-MPs were added into each culture dish for 2 h and kept in culture incubator in standard condition (37 °C with 5% CO_2_). And then, the cells were washed twice with ice-cold PBS and sent for fluorescent confocal imaging.

After HeLa cells (1 mL, 1 × 10^5^ cells/mL) were seeded in a 15 mm culture dish for 24 h, varying concentrations of EGCG (0 μM, 10 μM, 20 μM, or 40 μM) were added into each cell-adhered culture dish and incubated with HeLa cells for 24 h before detecting telomerase-induced fluorescence intensity change of AuNP-MPs with fluorescent confocal imaging.

### Intracellular telomerase activity-initiated Dox release

HeLa cells were seeded into the confocal dishes and cultured at 37 °C for 24 h with 5% CO_2_ in a humidified incubator. 100 μL 1.2 nM Dox-AuNP-MPs were added into confocal dishes to incubate with cells for different time periods. The cells were washed twice using ice-cold PBS before fluorescent confocal imaging.

HeLa, MCF-7, and L-02 cells were seeded into the individual culture dish for 24 h, respectively. 100 μL Dox-AuNP-MPs were added into each dish and incubated for 2 h. Cells were then washed twice with ice-cold PBS and imaged with confocal microscopy.

Different concentrations of EGCG (0 μM, 10 μM, 20 μM, or 40 μM) were incubated with HeLa cells for 24 h in confocal dish before detecting telomerase activity-triggered Dox release from Dox-AuNP-MPs with fluorescent confocal imaging.

### ICP-OES sample preparation and measurements

ICP-OES measurements were conducted on an Agilent 730 ICP-OES. Operating conditions of the ICP-OES are listed below: RF power: 1000 W; plasma Ar flow rate: 15 L/min; auxiliary gas flow rate: 1.5 L/min; nebulizer pressure: 200 kPa. AuNP-MPs and Dox-AuNP-MPs (4 nM) was incubated with HeLa, MCF-7 and L-02 cells in 6 well plates. After 2 h, all cells were washed three times with PBS buffer and then lysis buffer (200 µL) was added to the cells. The resulting cell lysate was digested overnight using 3 mL of HNO_3_ and 1 mL of H_2_O_2_. On the next day, 3 mL of aqua regia was added to be allowed to react for another 3 h. The final sample solution was measured by ICP-OES under the operating conditions described above.

### Flow cytometric analysis of fluorescence intensity generated by telomerase-responsive AuNP-MPs]

HeLa, MCF-7, A549, and L-02 cells were seeded into a 6-well plate at a concentration of 1 × 10^5^ cells/mL, respectively and cultured for 24 h. And then, 100 μL 1.2 nM AuNP-MPs were added into the 6-well plate and incubated at 37 °C for 2 h. Medium was removed, and the cells were washed twice with ice-cold PBS. Next, the cells were separated by trypsin and collected for flow cytometry to detect fluorescence intensity.

Various concentrations of EGCG (0 μM, 10 μM, 20 μM, or 40 μM) were added into the 6-well plate and incubated with HeLa cells for 24 h. 100 μL AuNP-MPs (12 nM) were added into the 6-well plate and incubated at 37 °C for 2 h. The culture medium was removed and the cells were washed twice by using ice-cold PBS. Finally, the cells were trypsinized and collected for flow cytometry to detect fluorescence intensity.

### The cytotoxicity assessment

HeLa and L-02 cells were seeded and cultured for 24 h in 96-well plates at a concentration of 1 × 10^4^ cells/mL. The cells were incubated with various concentrations of AuNPs, AuNP-MPs, or Dox-AuNP-MPs for 48 h. As a control, Dox was also incubated with cells with same concentration as AuNP-MPs. Later, the culture medium was removed and the cells were incubated with FBS-free culture medium for 48 h. MTT assay was performed as per the operational guidance of Kit. Absorbance was obtained using a microplate reader at a wavelength of 570 nm.

## Electronic supplementary material

Supplementary Information
