# Supplementary material for: Molecular imaging of telomerase and the enzyme activity-triggered drug release by using a conformation-switchable nanoprobe in cancerous cells
Source: Sci Rep. 2018 Nov 5;8:16341. doi: 10.1038/s41598-018-34670-7 (PMC6218543; doi:10.1038/s41598-018-34670-7)
Supplement: Supplementary file 1 — Supplementary Information [file 41598_2018_34670_MOESM1_ESM.docx]

**Supplementary Information**

Molecular imaging of telomerase and the enzyme activity-triggered drug release by using a conformation-switchable nanoprobe in cancerous cells

*Hai Shi,*^1^ *Tao Gao,*^2^ *Liu Shi,*^1^ *Tianshu Chen,*^2^ *Yang Xiang,*^1^ *Yuanyang Li,^3,^***and Genxi Li*^1,2^*^,^*^*^

^1^State Key Laboratory of Pharmaceutical Biotechnology and Collaborative Innovation Center of Chemistry for Life Sciences, Department of Biochemistry, Nanjing University, Nanjing 210093, P. R. China

^2^Center for Molecular Recognition and Biosensing, School of Life Sciences, Shanghai University, Shanghai 200444, P. R. China

^3^Department of Neurosurgery, Nanjing Integrated Traditional Chinese and Western Medicine Hospital, Affiliated with Nanjing University of Chinese Medicine, Nanjing 210014, P. R. China

The detailed DNA sequences used in study.

**Table S1.** The DNA sequences.

| Name | Sequence (5ʹ end to 3ʹ end) | Length (nt) |
| --- | --- | --- |
| HP-1 | SH-TTTTTTTATTAGGGTTAGGGTTTTTTTTTTTTTTTTTTTTTCCCTAACCCT(*-FAM*)AAACTGTTTTTTTCAGT | 68 |
| HP-2 | SH-TTTTTTTATTAGGGTTAGGGTTTTTTTTTTTTTTTTTTTTTCCCTAACCCT(*-FAM*)AATTTTTTTCAGT | 64 |
| HP-3 | SH- TTTTTTTAAAAGGGATAGGGTTTTTTTTTTTTTTTTTTTTTC CCTAT(*-FAM*)CCCTTTTTTTTTTCAGT | 64 |
| HP-1 | TTTTTTTATTAGGGTTAGGGTTTTTTTTTTTTTTTTTTTTTCCCTAACCCTAAACTGTTTTTTTCAGT | 64 |
| TS | AATCCGTCGAGCAGAGTT | 18 |
| CX | CCCTTACCCTTACCCTAACCCTTA | 24 |


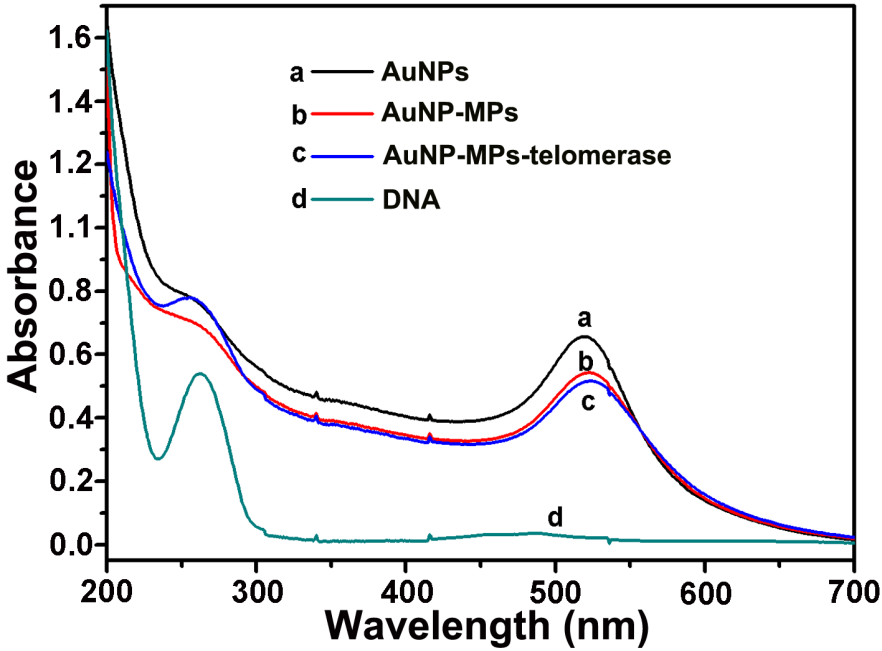


**Fig. S1** UV-vis spectra of bare AuNPs (a), AuNP-MPs in the absence (b) or presence (c) of telomerase, and DNA (d).


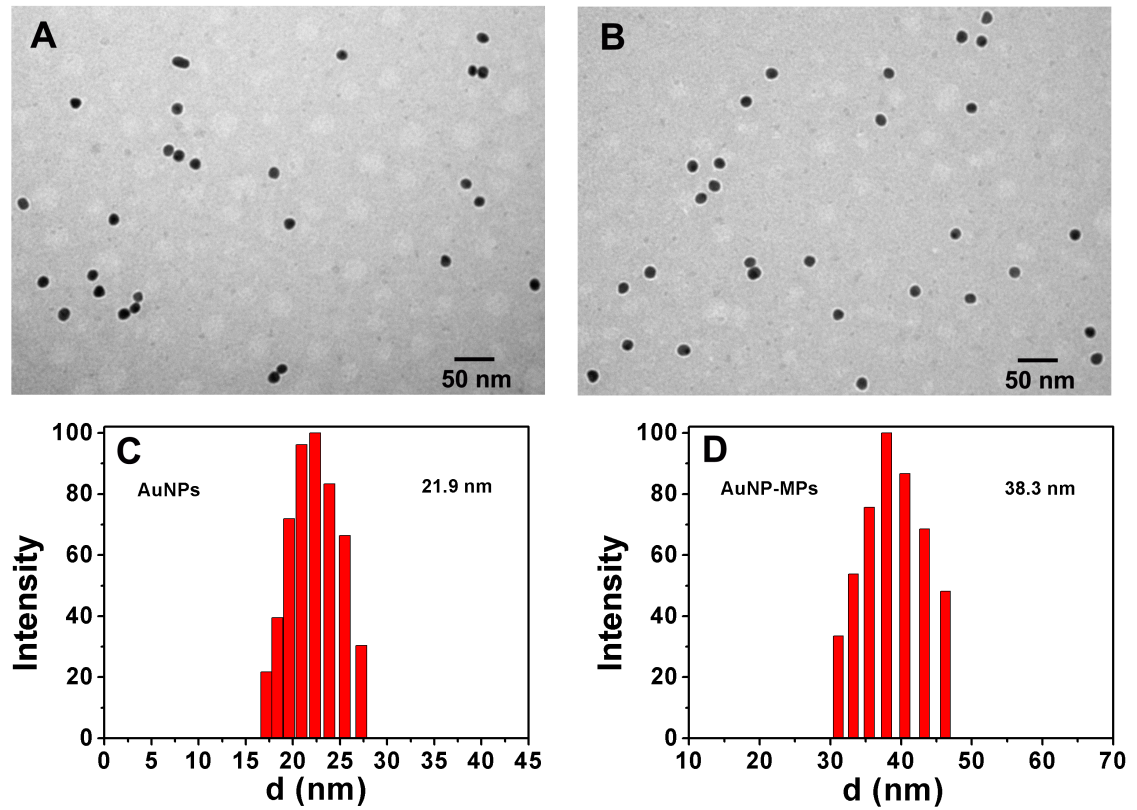


**Fig. S2** Characterization of the synthesized AuNP-MPs. (A) TEM image of the prepared AuNPs. (B) TEM image of the prepared AuNP-MPs. (C) DLS characterization of the synthesized AuNPs. (D) DLS characterization of the synthesized AuNP-MPs.

**
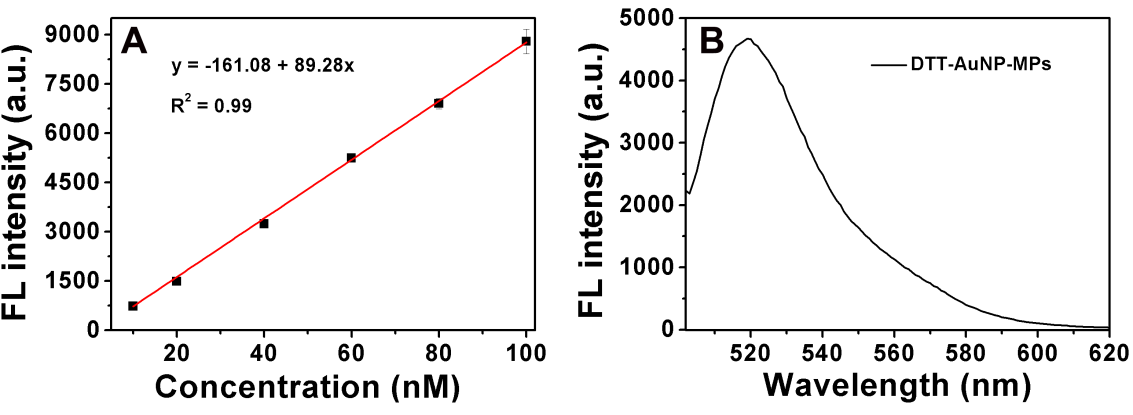
**

**Fig. S3** Quantitation of hairpin DNA number on one AuNP. (A) The linear relationship between the fluorescence intensity of various concentrations of hairpin DNA and the concentrations of hairpin DNA. (B) Florescence emission spectra of the released hairpin DNA from 2 nM AuNP-MPs. Error bars indicate standard deviation of triplicate tests.


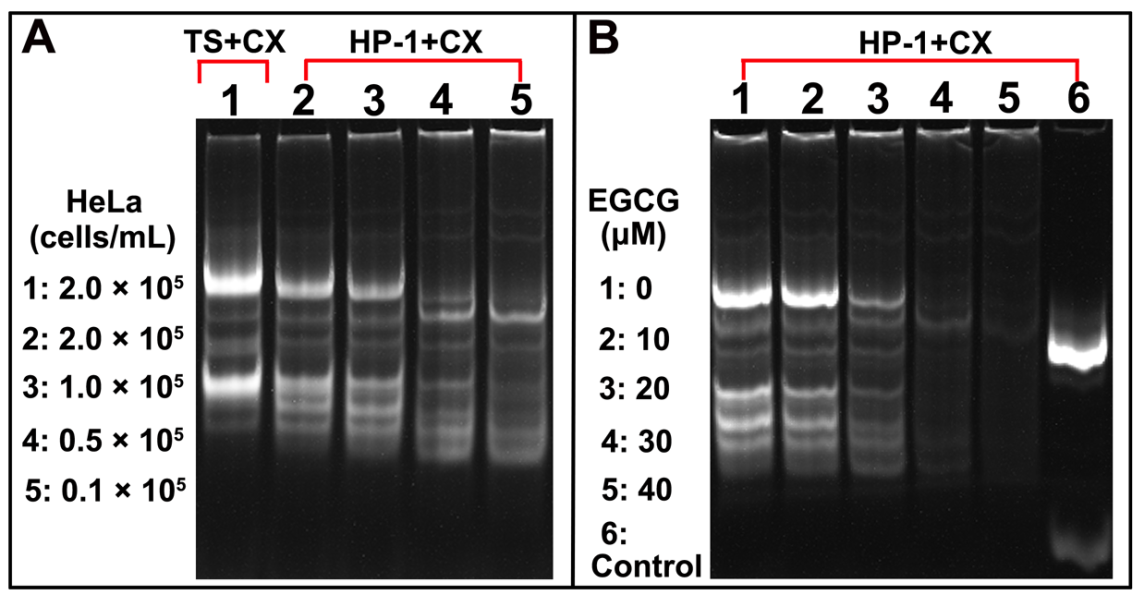


**Fig. S4** Telomeric repeat amplification protocol (TRAP) assay of telomerase amplification products using HP-1 as primer. (A) TRAP assay of telomerase amplification products produced by various concentrations of cell extracts. TS primer was chosen as the positive control. (B) TRAP assay of telomerase amplification products produced by various concentrations of EGCG-treated cell extracts, the concentration of telomerase extract was 2 × 10^5^ cells/mL. As a control, HP-1 was used directly as the template for PCR without telomerase amplification (Lane 6 in B).

**
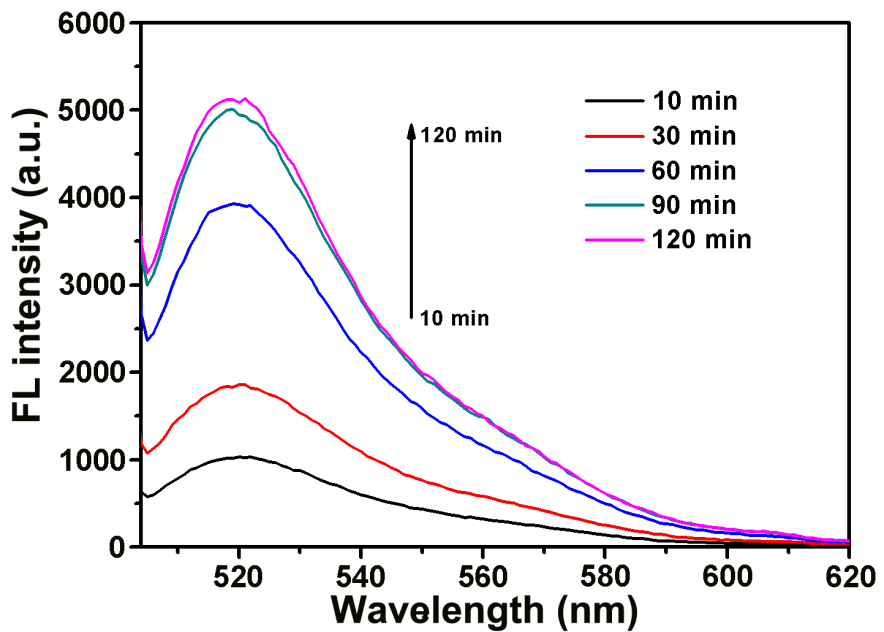
**

**Fig. S5** Fluorescence response of AuNP-MPs (4 nM) to telomerase elongation after incubation with dNTPs (400 µM) and telomerase extraction at 37 ºC for 10, 30, 60, 90, and 120 min.

**
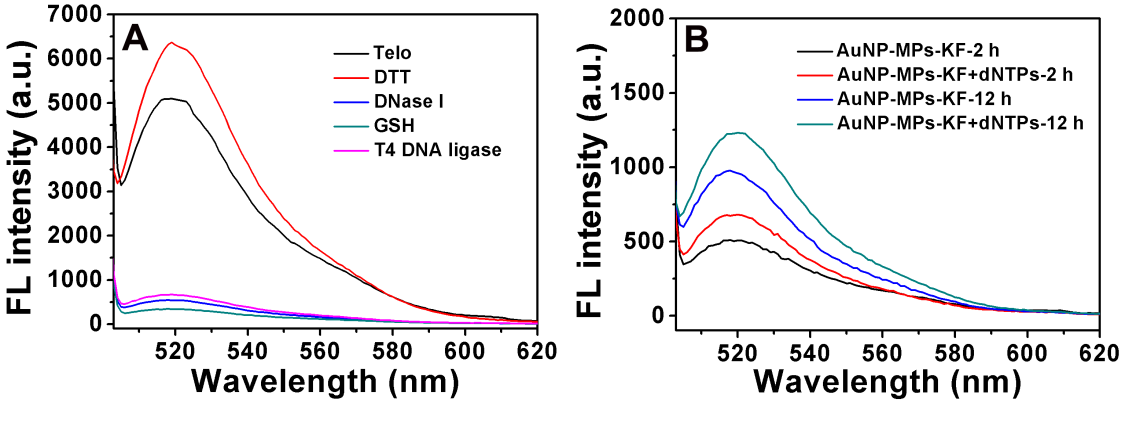
**

**Fig. S6** Stability of the AuNP-MPs under different situations. (A) Fluorescence spectra of AuNP-MPs (4 nM) after incubation with T4 DNA ligase, DNase I, GSH, DTT and telomerase (in the presence of dNTPs), respectively. (B) The fluorescent changes of AuNP-MPs after incubation with 4 U/mL DNA polymerase I (Klenow fragment) in the presence or absence of dNTPs.

**
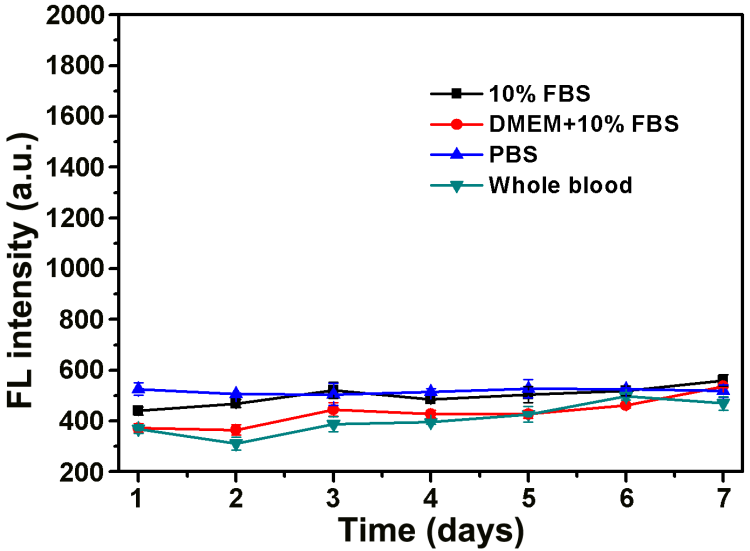
**

**Fig. S7** The fluorescence intensity of the AuNP-MPs dispersed in 10% FBS, DMEM + 10% FBS, PBS, whole blood for 1-7 days, respectively.

**
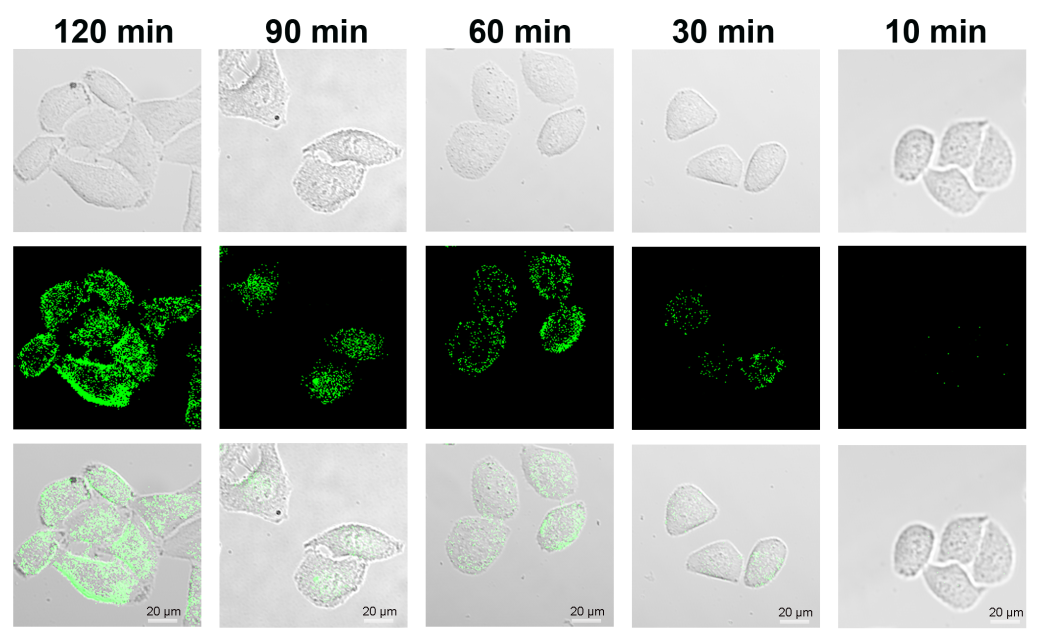
**

**Fig. S8** Confocal images of HeLa cells incubated with 1.2 nM AuNP-MPs for 10, 30, 60, 90, and 120 min, respectively.

**
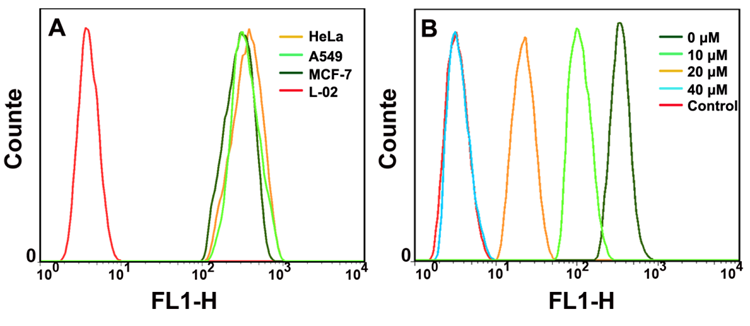
**

**Fig. S9** (A) Flow cytometric detection of cancerous cells and normal cells after incubation with the nanoprobe for 2 h. (B) Flow cytometric detection of HeLa cells after treatment with EGCG (0 μM, 10 μM, 20 μM, or 40 μM) for 24 hours, then incubated with 100 μL AuNP-MPs (1.2 nM) for 2 h.

**
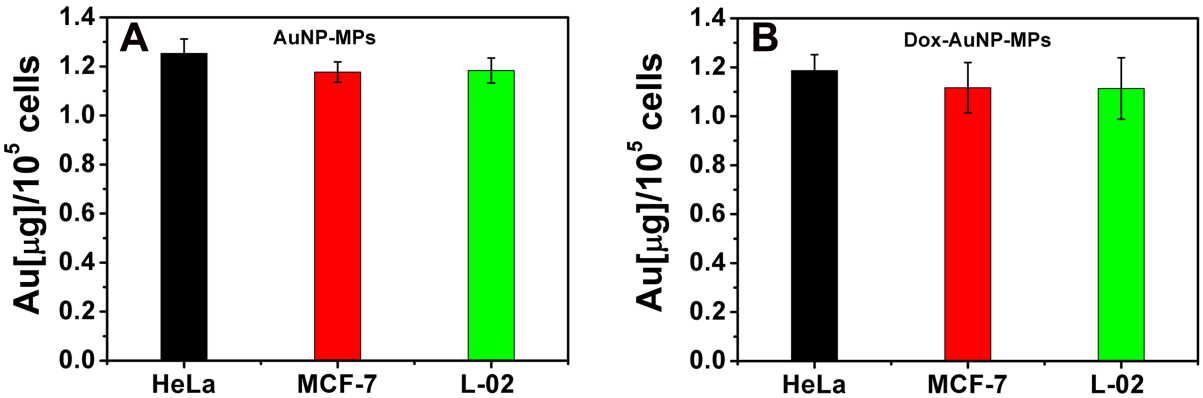
**

**Fig. S10** Quantification of the amount of gold present in cells with inductively coupled plasma optical emission spectroscopy (ICP-OES) in each HeLa, MCF-7 and L02 cell. (A) The cells were incubated with 4 nM AuNP-MPs for 2 h. (B) The cells were incubated with 4 nM Dox-AuNP-MPs for 2 h. Error bars indicate standard deviation of triplicate tests.


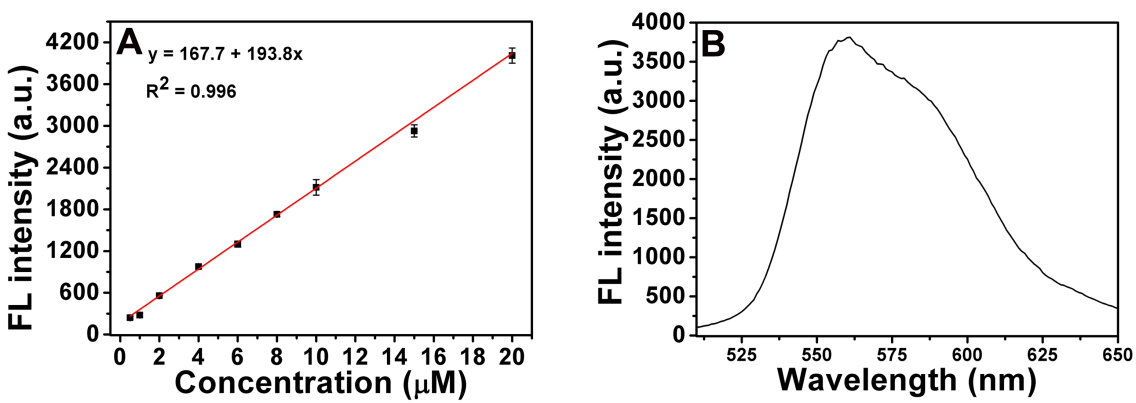


**Fig. S11** Quantitation of Dox amount on one AuNP-MP. (A) The linear relationship between the fluorescence intensity of various concentrations of Dox and the concentrations of Dox. (B) Florescence emission spectra of the supernatant solution after overnight incubation of Dox with AuNP-MPs. Error bars indicate standard deviation of triplicate tests.

**
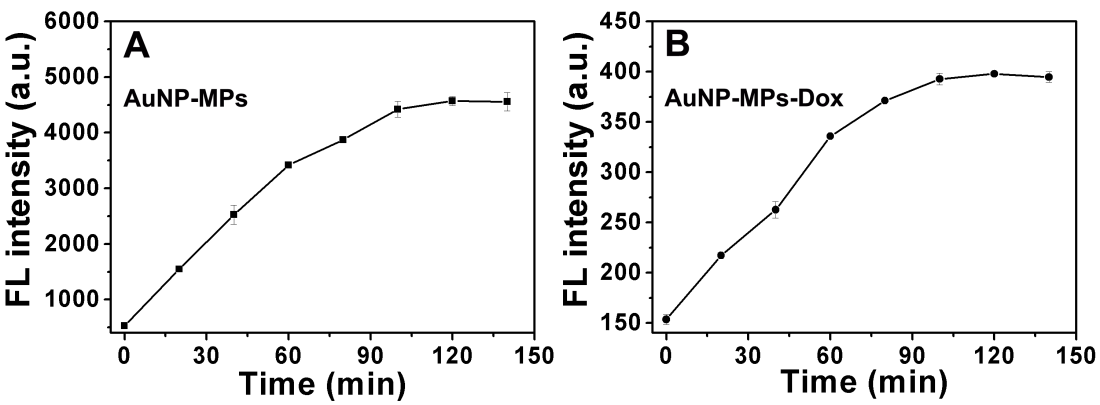
**

**Fig. S12** The fluorescence intensity of released FAM and Dox from Dox-AuNP-MPs (4 nM) with telomerase extracts and dNTPs.

**

**

**Fig. S13** The fluorescence intensity of released Dox from Dox-AuNP-MPs (4 nM) and Dox-AuNP-MPs3 (4 nM) after incubation with different concentration of telomerase extracts and 400 μM dNTPs for 2 h.

**
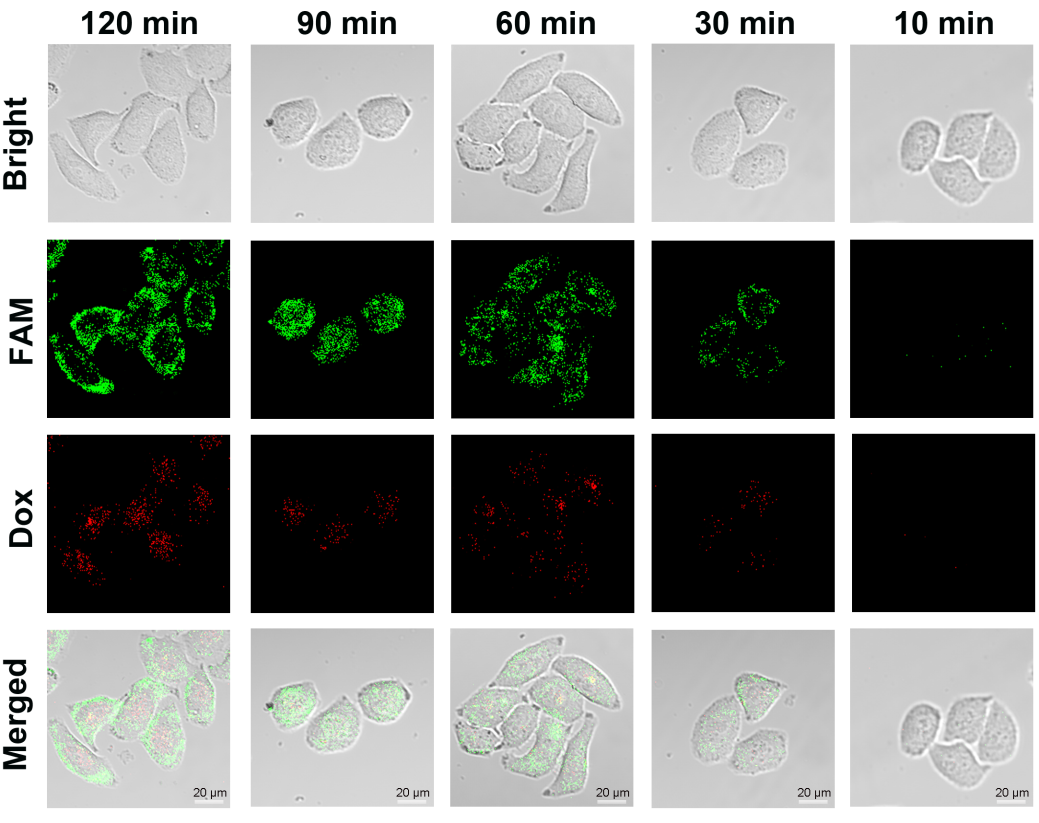
**

**Fig. S14** Confocal images of HeLa cells incubated with 100 uL Dox-AuNP-MPs (1.2 nM) for 10, 30, 60, 90, and 120 min, respectively.

**
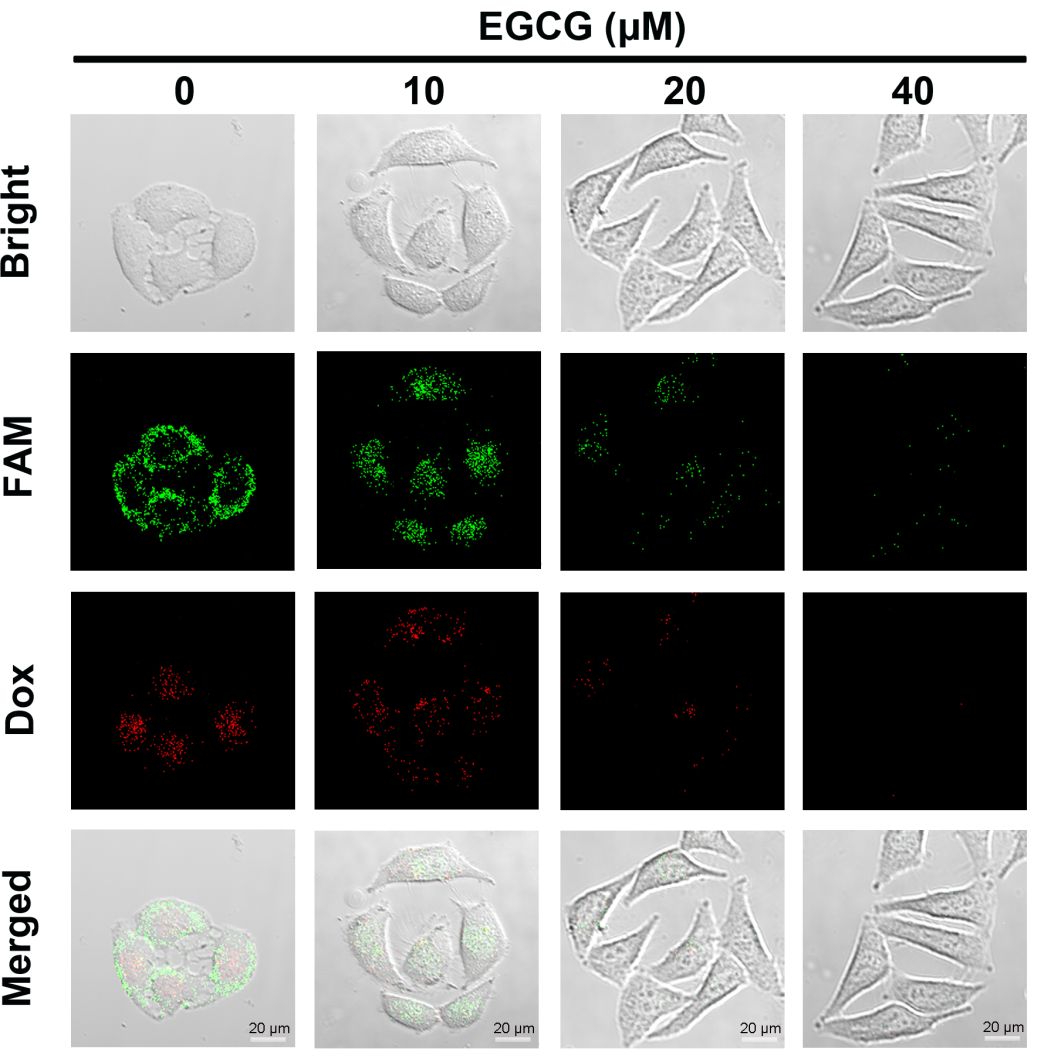
**

**Fig. S15** Confocal images of HeLa cells after incubation with different concentrations of EGCG (0 μM, 10 μM, 20 μM, or 40 μM) for 24 h, then incubated with 100 μL Dox-AuNP-MPs (1.2 nM) for 2 h.

**
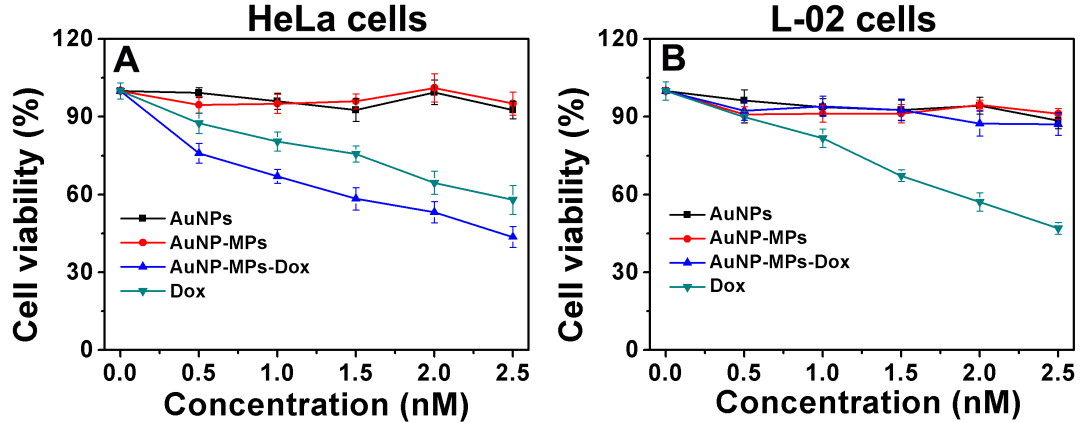
**

**Fig. S16** In vitro cytotoxicity of Dox, AuNPs, AuNP-MPs, and Dox-AuNP-MPs to HeLa cells and L-02 cells. The used drug or nanoprobes were varied from 0 to 2.5 nM. Data represent mean ±SD (n=6).

**Table S2**. Comparison of the limit of detection (LOD) for the as-proposed method and the reported methods for the detection of telomerase activity

| Strategy | Detection mode | LOD | reference |
| --- | --- | --- | --- |
| ECL sensor using G-quadruplex and luminol modified AuNPs | ECL | 148 HL-60 cells | 1 |
| Au film-based assay | SPR | 18 293T cells/μL | 2 |
| Exonuclease I manipulating primer-modified AuNPs | Colorimetry | 29 HL-60 cells/mL | 3 |
| Platinum nanoparticles encapsulated metal–organic frameworks | electrochemistry | 100 HeLa cells/mL | 4 |
| Ratiometric sensing based on structure-switching DNA | FRET | 33 HeLa cells or 41 MCF-7 cells | 5 |
| TRAP assay | Radioactive signal | 1293 cells | 6 |
| Conformation-switchable nanoprobe | Fluorescence | 59 HeLa cells/mL | This work |

1. Zhang, H. R. *et al*. Visual electrochemiluminescence detection of telomerase activity based on multifunctional Au nanoparticles modified with G-quadruplex deoxyribozyme and luminol. Chem Commun 50, 12575−12577, (2014).
2. Sharon, E., Freeman, R., Riskin, M., Gil, N., Tzfati, Y. & Willner, I. Optical, electrical and surface plasmon resonance methods for detecting telomerase activity. Anal Chem 82, 8390−8397, (2010).
3. Zhang, L., Zhang, S. J., Pan, W., Liang, Q. C. & Song, X. Y. Exonuclease I manipulating primer-modified gold nanoparticles for colorimetric telomerase activity assay. Biosens Bioelectron 77, 144−148, (2016).
4. Ling, P. H., Lei, J. P., Jia, L. & Ju, H. X. Platinum nanoparticles encapsulated metal–organic frameworks for the electrochemical detection of telomerase activity. Chem Commun 52, 1226−1229, (2016).
5. Yang, X. J., Zhang, K., Zhang, T. T., Xu, J. J. & Chen, H. Y. Reliable förster resonance energy transfer probe based on structure-switching DNA for ratiometric sensing of telomerase in living cells. Anal Chem 89, 4216−4222, (2017).
6. Kim, N. W. & Wu, F. Advances in quantification and characterization of telomerase activity by the telomeric repeat amplification protocol (TRAP). Nucleic Acids Res 25, 2595−2597, (1997).
